# Supplementary material for: Detecting initial correlations via correlated spectroscopy in hybrid quantum systems
Source: Sci Rep. 2021 Oct 20;11:20718. doi: 10.1038/s41598-021-99718-7 (PMC8528928; doi:10.1038/s41598-021-99718-7)
Supplement: Supplementary file 1 — Supplementary Information. [file 41598_2021_99718_MOESM1_ESM.pdf]

Detecting Initial Correlations via Correlated Spectroscopy in Hybrid Quantum Systems

Parth Jatakia[1,2,*], Sai Vinjanampathy[1,3], and Kasturi Saha[4]

[1]Department of Physics, Indian Institute of Technology Bombay, Mumbai , India
[2]Department of Electrical Engineering, Princeton University, Princeton, New Jersey 08540, USA
[3]Centre for Quantum Technologies, National University of Singapore, Singapore
[4]Solid State Device Group, Department of Electrical Engineering, Indian Institute of Technology Bombay, Mumbai , India
[*]pjatakia@princeton.edu

1 Conditional Preparation as a means to probe specific correlations

Post-conditional preparation the system-environment state is $|0\rangle\langle 0| \otimes \{\mathrm{Tr}_s[(|m\rangle\langle m| \otimes \mathbb{I}_E)\chi] + \tau\}$ as defined in Eqn.1 of the main paper. The correlation matrix χ can be written in form of system states ($|i_s\rangle$) and environment states ($|i_e\rangle$) as

$$\chi = \sum_{\substack{i_s,j_s\\ i_e,j_e}} \chi_{\substack{i_s,j_s\\ i_e,j_e}} |i_s i_e\rangle\langle j_s j_e| \,. \tag{1}$$

Substituting back in $P := (|m\rangle\langle m| \otimes \mathbb{I}_E)\cdot\chi$ gives

$$P = \sum_{\substack{i_s,j_s\\ i_e,j_e}} \chi_{\substack{i_s,j_s\\ i_e,j_e}} (|m\rangle\langle m| \otimes \mathbb{I}_E)(|i_s i_e\rangle\langle j_s j_e|)\,, \tag{2}$$

where P is post entanglement breaking channel part of environment which remembers the initial correlation. Partial tracing over the system gives

$$\mathrm{Tr}_s(P) = \sum_{\substack{i_s,j_s\\ i_e,j_e}} \chi_{\substack{i_s,j_s\\ i_e,j_e}} \langle j_s|m\rangle \langle m|i_s\rangle |i_e\rangle\langle j_e| \,. \tag{3}$$

Thus for $\chi_{i,j}$ (where $i = i_s N_s + i_e,\ j = j_s N_s + j_e$ and N_s is the Hilbert space dimension of the system) to be retained after conditional preparation $\langle m|i_s\rangle$ and $\langle j_s|m\rangle$ should not be zero. Hence the projection operator $|m\rangle\langle m|$ should not be orthogonal to $|i_s\rangle$ and $|j_s\rangle$.

2 Conditions for the signal to not register a finite $\chi_{i,j}$

For $\chi_{i,j}$ independent signal, the derivative of signal with respect to $\chi_{i,j}$ should be zero. If the final measurement operator is $|n\rangle\langle n|$, the measured signal is

$$N(t_1,t_2) = Tr\left[(|n\rangle\langle n| \otimes \mathbb{I}_E)\,\rho^{SE}(t_1,t_2)\right]. \tag{4}$$

$|n\rangle\langle n|$ and $\rho_{SE}(t)$ can be written in their element-wise format giving $N(t_1,t_2)$ as

$$= \mathrm{Tr}\left(\sum_{k,l} n_{kl}\, |k\rangle\langle l| \otimes \mathbb{I}_E \cdot \sum_{\substack{i_s,j_s\\ i_e,j_e}} \rho^{SE}_{\substack{i_s j_s\\ i_e j_e}}(t_1,t_2)\, |i_s i_e\rangle\langle j_s j_e|\right). \tag{5}$$

On simplification gives $N = \sum_k n_{kk} \sum_{i_e} \rho^{SE}_{kki_ei_e}(t_1,t_2)$ where, $\rho^{SE}(t_1,t_2)$ can be written using system-environment unitary evolution operators as

$$\rho^{SE}(t_1,t_2) = e^{-\imath H t_2} e^{-\imath \frac{\pi}{2} A} e^{-\imath H t_1} \rho_{SE}(0) e^{\imath H t_1} e^{\imath \frac{\pi}{2} A} e^{\imath H t_2}. \tag{6}$$

Simply can be written as $\mathscr{U}\cdot\rho_{SE}(0)\cdot\mathscr{U}^\dagger$ for now, where $\mathscr{U}$ describes the complete unitary evolution, H is the system-environment total Hamiltonian and $\hat{A}$ is a rotation operator. Here $\rho^{SE}(0)$ is given by the element expansion in Eqn. 2.

Substituting back, differentiating with $\chi_{i_s^{'},j_s^{'},i_e^{'},j_e^{'}}$ and on simplifying,

$$\frac{\partial N}{\partial \chi_{\substack{i_s^{'},j_s^{'}\\ i_e^{'},j_e^{'}}}} = \sum_{k,i_e} n_{kk} m_{j_s}^{'} m_{i_s}^{'} \left[\mathscr{U} \cdot \left|0,i_e^{'}\right\rangle\left\langle 0,j_e^{'}\right| \cdot \mathscr{U}^{\dagger}\right]_{\substack{k,k,\\ i_e,i_e}}, \tag{7}$$

where $m_{i_s}^{'}$ $(m_{j_s}^{'}) = \left\langle m \middle| i_s^{'} \right\rangle$ $(\left\langle m \middle| j_s^{'} \right\rangle)$. If the projection operator $|m\rangle\langle m|$ is not orthogonal to $\left| i_s^{'} \right\rangle$ and $\left| j_s^{'} \right\rangle$, then $m_{i_s}^{'}$,$m_{j_s}^{'}$ are non zero. For signal to be independent Eqn. 7 should be zero. This will lead to

$$\sum_{k,i_e} n_{kk} \left[\mathscr{U} \cdot \left|0,i_e^{'}\right\rangle\left\langle 0,j_e^{'}\right| \cdot \mathscr{U}^{\dagger}\right]_{k,k,i_e,i_e} = 0. \tag{8}$$

Thus for $\chi_{i^{'},j^{'}}$ independence, there should be no population change in $|n\rangle\langle n|$ due to $\left|0,i_e^{'}\right\rangle\left\langle 0,j_e^{'}\right|$ under PrePSy. Hence there should exist seperate Hilbert subspaces consisting of $\left|0,i_e^{'}\right\rangle\left\langle 0,j_e^{'}\right|$ and $|n\rangle\langle n|$ respectively under action of Hamiltonian (H) and $\pi/2$ rotation due to $\hat{A}$.

3 Characterizing initial correlation using only one-dimensional spectroscopy

One-dimensional spectroscopy for PrePSy is convenient for quickly observing and measuring the initial correlations due to the reduced experimental effort. Only having single-dimensional spectroscopy implies the computational complexity is reduced, and hence parameters of more complex systems can be obtained from fitting the data. The drawback of one-dimensional spectroscopy is the ambiguity in accurately calculating the correlations and Hamiltonian as the output of PrePSy, which is no more unique and is shown in conclusion.

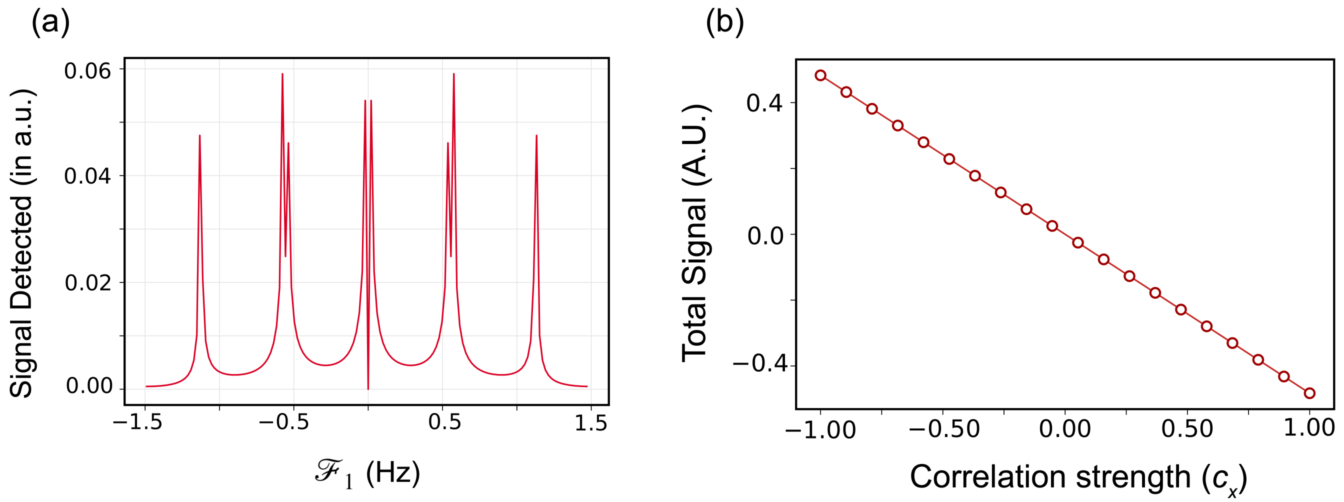

Supplementary Figure 1. One dimensional PrePSy applied on the toy model. (a) 1D Date of PrePSy with parameters as $c_x = 1$, $\lambda^{xx} = 4Hz$, $\lambda^{yy} = 3Hz$ and $\lambda^{zz} = 3.5Hz$. (b) Plot of the variation in total signal strngth vs the initial correlation c_x.

For the toy model described in Section A.a, one-dimensional PrePSy was carried with the same parameters. The results obtained are shown in Figure 1, where the initial projection operators ($|x\rangle\langle x|$ and $|-x\rangle\langle -x|$) and the measurement operator ($|x\rangle\langle x|$) are kept the same. As the configuration of PrePSy is identical, the positions of the peaks will not change. However, the shapes of the peaks will change as the spectroscopy process is different. The presence of peaks indicates the presence of correlations.

Similar to the two-dimensional case, the total correlation varies linearly with the amount of initial correlation present between the system and the environment, as seen in Figure 1. Hence the method to measure correlation remains the same as discussed in Section 3.B

4 Adiabatic elimination of the cavity

The method to obtain the effective Hamiltonian by adiabatic elimination under dispersive coupling regime is based on time-averaging of the Hamiltonian[1]. A perturbative Hamiltonian with a harmonic time dependence is of the form

$$\hat{H}_I(t) = \sum_{n=1}^{N} \hat{h}_n \exp(-i\omega_n t) + \hat{h}_n^\dagger \exp(i\omega_n t), \tag{9}$$

where N is the total number of different harmonic terms making up the interaction Hamiltonian and $\hat{h}_n$ are the operators defining the interactions. The effective Hamiltonian obtained by letting the fast evolving terms average to zero, is

$$\hat{H}_{eff}(t) = \sum_{m,n=1}^{N} \frac{1}{\hbar\overline{\omega}_{mn}} \left[\hat{h}_m^\dagger, \hat{h}_n\right] \exp(i[\omega_m - \omega_n]t), \tag{10}$$

where $\overline{\omega}_{mn}$ is the harmonic average of ω_m and ω_n, viz.,

$$\frac{1}{\bar{\omega}_{mn}} = \frac{1}{2}\left(\frac{1}{\omega_m} + \frac{1}{\omega_n}\right). \tag{11}$$

Thus using this method the lab frame interaction Hamiltonian of just "N" NVs interacting with the cavity field is as follows

$$\hat{H} = \sum_{i}^{N} \left[g_i\left(a^\dagger |0_i\rangle\langle e_i| e^{i\Delta t} + a|e_i\rangle\langle 0_i| e^{-i\Delta t}\right)\right]. \tag{12}$$

Applying Eqn. 10 on the above Hamiltonian to get the effective Hamiltonian as

$$\begin{aligned}\hat{H}_{\text{eff}} = &\sum_{\substack{i,j\\ i\neq j}}^{N} \frac{\hbar g_i g_j}{\Delta} \left|0_i e_j\right\rangle\left\langle e_i 0_j\right| + \left|e_i 0_j\right\rangle\left\langle 0_i e_j\right| \\ &+ \sum_{i=1}^{N} \frac{\hbar g^2}{\Delta} \left(|e_i\rangle\langle e_i| - |0_i\rangle\langle 0_i|\right)\left(2\hat{a}^\dagger\hat{a} - 1\right).\end{aligned} \tag{13}$$

The second term is the quantum Stark term, which is eliminated by firstly using a laser coupled to the same transition and secondly by assuming that the cavity is initially in the vacuum state. Additionally, a laser with zero detuning and Rabi frequency Ω is coupled to $|1_i\rangle \leftrightarrow |e_i\rangle$ transition to generate Raman-like transition. Thus the Hamiltonian is

$$H_{\text{adiab}} = \sum_{\substack{i,j\\ i\neq j}}^{N} \left[\frac{g_i g_j}{\Delta} \left|e_i 0_j\right\rangle\left\langle 0_i e_j\right|\right] + \sum_{i}^{N} \left[\Omega_i\left(|e_i\rangle\langle 1_i| + |1_i\rangle\langle e_i|\right)\right]. \tag{14}$$

Applying a change of basis $\left|+_{ij}\right\rangle = \left(\left|e_i 0_j\right\rangle + \left|0_i e_j\right\rangle\right)/\sqrt{2}$, $\left|-_{ij}\right\rangle = \left(\left|e_i 0_j\right\rangle - \left|0_i e_j\right\rangle\right)/\sqrt{2}$. The Hamiltonian in the new basis can be seperated into two non-interacting subspaces. Choosing the subspace $\{|10\rangle, |+\rangle, |-\rangle, |01\rangle\}$ and assuming the initial state to be in the subspace, the other subspace can be ignored. The Hamiltonian in this subspace is

$$\begin{aligned}H = &\sum_{\substack{i,j\\ i\neq j}}^{N} \left|+_{ij}\right\rangle\left\langle +_{ij}\right| (\Theta_{i,j}) - \left|-_{ij}\right\rangle\left\langle -_{ij}\right| (\Theta_{i,j}) \\ &+ \sum_{i}^{N} \Lambda_i |+\rangle\langle 10| + \Lambda_i |-\rangle\langle 10| + H.C.,\end{aligned} \tag{15}$$

where $\Theta_{i,j} = g_i g_j/\Delta$ and $\Lambda_i = \Omega_i/\sqrt{2}$. The schematic diagram of the interaction is given by Figure 4.C. of main paper. Applying adiabatic elimination given by Eqn. 10 again, the effective Hamiltonian obtained is given by Eqn. 11 in main paper.

References

1. James, D. & Jerke, J. Effective hamiltonian theory and its applications in quantum information. *Can. J. Phys.* **85**, 625–632 (2007).
